# Application of individualized osteotomy and registration guide plate in temporomandibular joint gap arthroplasty: a pilot clinical study

**DOI:** 10.3389/froh.2025.1655362

**Published:** 2025-09-02

**Authors:** Jianfei Zhang, Jian Sun, Tengfei Jiang, Guokai Zhou, Yuan Tian, Liang Xia, Wenbin Zhang

**Affiliations:** 1 Department of Oral & Cranio-maxillofacial Surgery, Shanghai Ninth People’s Hospital, College of Stomatology, Shanghai Jiao Tong University School of Medicine, National Clinical Research Center for Oral Diseases, Shanghai Key Laboratory of Stomatology & Shanghai Research Institute of Stomatology, Shanghai, China; 2 Department of Stomatology, People’s Hospital of Xiangyun Affiliated to Dali University, Dali, Yunnan, China; 3 Institute of Molecular Medicine and Shanghai Key Laboratory for Nucleic Acid Chemistry and Nanomedicine, State Key Laboratory of Systems Medicine for Cancer, Renji Hospital, School of Medicine, Shanghai Jiao Tong University, Shanghai, China

**Keywords:** individualized guide, TMJ arthroplasty, ankylosis, craniomaxillofacial surgery, navigation-assisted surgery

## Abstract

**Introduction:**

Temporomandibular joint (TMJ) ankylosis severely compromises mandibular mobility and overall oral function. Gap arthroplasty remains the standard surgical treatment. However, the accuracy of navigation-assisted procedures is frequently limited by anatomical registration challenges, particularly when relying on dental surface matching.

**Methods:**

This single-center prospective study enrolled 30 patients with unilateral bony TMJ ankylosis. Participants were randomly assigned to either a dental surface registration group (*n* = 18) or a guide plate registration group (*n* = 12). The patient-specific guide plate integrated both osteotomy slots and fiducial markers. Primary outcomes included registration time and target registration error (TRE). Secondary outcomes were operative time, mouth opening at three months, and perioperative complications.

**Results:**

The guide plate group achieved significantly shorter registration times (56.23 ± 11.33 s; 95% CI: 49.35–63.11) compared with the dental registration group (935.03 ± 85.40 s; 95% CI: 894.55–975.51; *P* < 0.001). TRE was also significantly lower in the guide plate group (0.69 ± 0.10 mm; 95% CI: 0.62–0.76) than in the dental registration group (2.82 ± 0.45 mm; 95% CI: 2.60–3.04; *P* < 0.001). Average operative time was reduced in the guide plate group (124.63 ± 5.39 min; 95% CI: 121.44–127.82) compared with the dental group (134.31 ± 12.76 min; 95% CI: 128.10–140.52; *P* = 0.009). Postoperative mouth opening at three months was comparable between groups (31.5 ± 4.23 mm; 95% CI: 28.94–34.06 vs. 31.33 ± 3.34 mm; 95% CI: 29.65−33.01; *P* = 0.905). No major intraoperative or postoperative complications were observed.

**Discussion:**

Integration of an individualized osteotomy and registration guide plate significantly improved intraoperative efficiency and spatial accuracy in TMJ gap arthroplasty without compromising functional outcomes. These pilot findings support the clinical feasibility of the guide plate system, though larger multicenter studies and evaluation of inter-operator variability are required for broader validation.

## Introduction

Temporomandibular joint (TMJ) ankylosis is a pathological condition characterized by the fusion of the mandibular condyle to the temporal bone, leading to functional limitations such as reduced mouth opening, impaired mastication, speech difficulties, and psychosocial distress (1). The etiology of TMJ ankylosis is multifactorial, with trauma, infection, systemic inflammatory disorders, and iatrogenic factors being common contributors. In children and young adults, ankylosis is particularly detrimental, as it may impede mandibular growth and facial symmetry, compounding long-term functional and aesthetic consequences (2, 3).

Gap arthroplasty remains the cornerstone surgical approach for managing TMJ bony ankylosis. The primary goals of surgical intervention are to restore mandibular mobility, prevent re-ankylosis, protect vital adjacent structures, and optimize functional and cosmetic outcomes. However, TMJ surgery is inherently complex due to the joint's deep anatomical location, limited access, and proximity to critical neurovascular structures such as the internal maxillary artery, facial nerve, and dura mater. This anatomical complexity mandates high surgical precision, especially during osteotomy and recontouring of the glenoid fossa (2).

In recent years, digital surgical technologies, including computer-assisted navigation systems, have been increasingly applied to craniofacial procedures to enhance surgical precision and reproducibility (4–6). Navigation systems offer real-time visual guidance by correlating preoperative imaging with intraoperative anatomy. The clinical success of these systems, however, is highly dependent on the accuracy of registration—the process by which the patient's physical anatomy is mapped to digital imaging datasets. Inaccurate registration propagates into target registration error (TRE), which may compromise surgical safety and outcome reliability (7, 8). Given the anatomically constrained and neurovascularly complex nature of the TMJ region, even minimal deviations in registration accuracy during gap arthroplasty may substantially elevate the risk of iatrogenic injury to adjacent critical structures.

The literature generally considers a TRE of less than 2 mm acceptable for clinical navigation (9). Exceeding this threshold, particularly in TMJ procedures, may directly affect the accuracy of osteotomies and the protection of adjacent tissues. Two principal registration techniques are commonly employed in craniofacial navigation, surface matching and fiducial-based registation. Surface matching using dental morphology or facial contours is non-invasive but susceptible to error from intraoral humidity, probe angulation, and soft tissue dynamics (8, 10). Fiducial-based methods provide better accuracy but often rely on bone-anchored screws or external frames, raising concerns regarding invasiveness and complexity (8, 11, 12). Moreover, In TMJ surgery a common limitation is the long spatial distance between reference points and the surgical site, which can amplify registration error due to geometric propagation (13, 14).

To overcome these challenges, we propose an individualized osteotomy and registration guide plate system designed specifically for TMJ gap arthroplasty. This dual-purpose device integrates pre-planned osteotomy trajectories and embedded fiducial markers into a patient-specific 3D-printed surgical guide. By anchoring the guide plate on stable and anatomically proximal landmarks, namely the zygomatic arch and temporal bone, the system minimizes the fiducial-to-target distance and provides a rigid platform for accurate and reproducible registration. This design may offer a practical solution for improving intraoperative navigation accuracy and reducing operator-dependent variability in TMJ surgery. Rather than replacing current standards, it aims to complement them by addressing the limitations of existing registration techniques.

This study aims to evaluate the feasibility, accuracy, and clinical utility of the individualized guide plate system in TMJ ankylosis surgery. Through a prospective comparative trial, we assess its impact on registration accuracy, operative time, surgical consistency, and postoperative functional outcomes relative to conventional dental surface registration. Our hypothesis is that the guide plate system will significantly reduce TRE and streamline intraoperative workflows, thereby enhancing surgical safety and precision. If successful, this system could improve accuracy and efficiency in digital TMJ surgery.

## Materials and methods

This prospective, controlled clinical trial was conducted at the Department of Oral and Craniomaxillofacial Surgery, Shanghai Ninth People's Hospital, Shanghai Jiao Tong University School of Medicine, from January 2021 to October 2024. The protocol was approved by the Institutional Review Board (IRB), and all patients signed informed consent forms before participation. Ethical principles outlined in the Declaration of Helsinki were strictly observed.

### Patient selection and baseline characteristics

**Figure 1:**
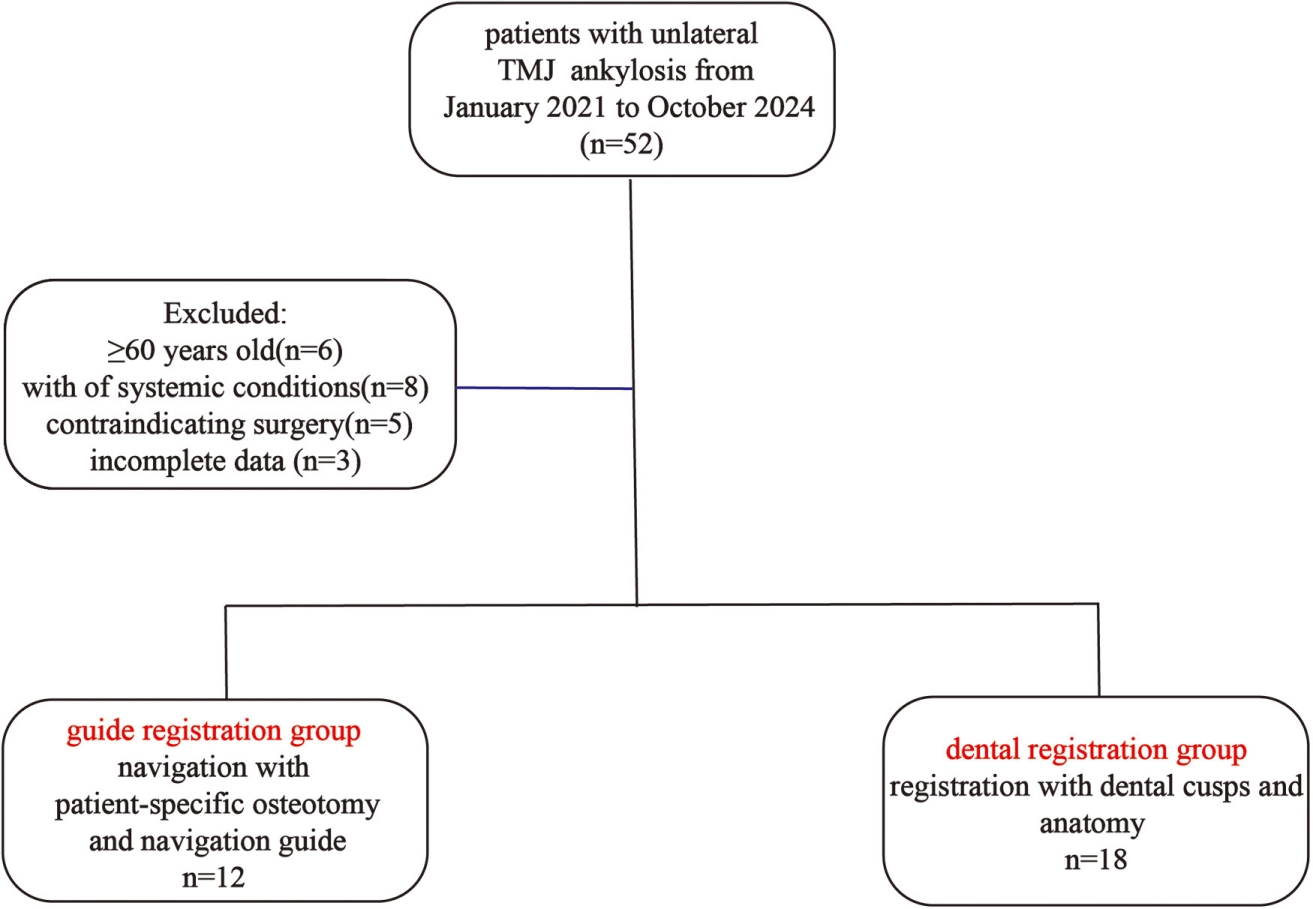
Schematic analysis of patients with **unilateral** TMJ ankylosis undergoing **navigation-assisted** arthroplasty using either guide plate registration or dental surface registration.

Thirty patients with unilateral bony ankylosis of the temporomandibular joint (TMJ) were enrolled after selection and randomly assigned to two groups: the guide plate registration group (*n* = 12) and the dental surface registration group (*n* = 18). Inclusion criteria were: 1. confirmed diagnosis of unilateral bony TMJ ankylosis through radiologic and clinical evaluation, 2. no previous TMJ surgeries, 3. adequate dental condition for intraoperative scanning, and 4. no systemic comorbidities that would contraindicate general anesthesia. Exclusion criteria included: patients over 60 years of age (*n* = 6), presence of uncontrolled systemic conditions (e.g., poorly controlled diabetes or cardiovascular disease) or physical status precluding safe anesthesia (*n* = 8), refusal or inability to undergo surgery (*n* = 5), and incomplete data due to loss to follow-up or missing imaging/surgical documentation (*n* = 3) (Figure 1).

The unequal group sizes were the result of block randomization stratified by age and ankylosis side. A greater number of patients were allocated to the dental registration group, which reflected the institutional standard of care at the time of study initiation.

Demographic comparison showed no statistically significant differences in age (33.91 ± 6.60 vs. 33.06 ± 8.82 years, *P* = 0.779) or gender distribution (Male: 5 vs. 14; Female: 7 vs. 4; *P* = 0.063). Ankylosis was evenly distributed between left and right sides across groups (Left: 58.3% vs. 50%; *P* = 0.654), ensuring comparability between cohorts.

All patients underwent preoperative high-resolution craniofacial CT scanning (1.25 mm slice thickness) using GE Healthcare systems. The Digital Imaging and Communications in Medicine (DICOM) files were imported into ProPlan CMF 3.0 (Materialise, Belgium) for 3D model reconstruction and virtual simulation of TMJ gap arthroplasty. The simulated osteotomy aimed to establish a 10 mm gap between the glenoid fossa and mandibular ramus. The planning stage included virtual removal of the ankylotic segment and trajectory analysis for safe osteotomy paths avoiding critical neurovascular structures.

### Guide plate design and fabrication

**Figure 2:**
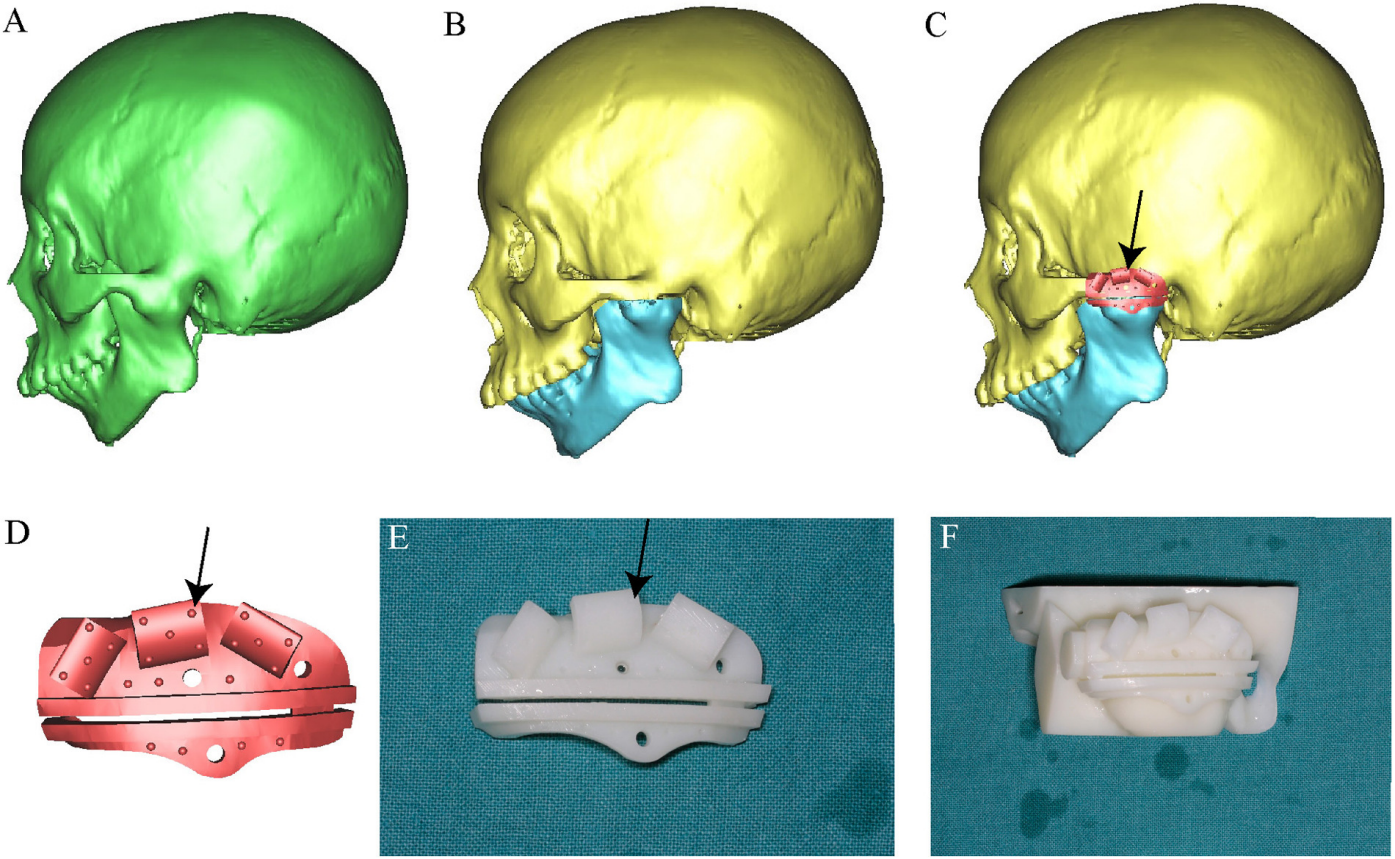
Virtual surgical planning and fabrication of a patient-specific osteotomy and registration guide plate. **(A)** Three-dimensional CT reconstruction reveals the ankylotic region of the temporomandibular joint. **(B)** Virtual simulation of the arthroplasty procedure. **(C)** Digital design of a customized osteotomy and registration guide plate tailored to the patient's anatomy. **(D)** Detailed view of the guide, featuring a single osteotomy slot, two screw holes for fixation, and three strategically distributed registration wings to ensure accurate alignment. **(E)** Additive manufacturing of the patient-specific guide. **(F)** The guide fits precisely in the TMJ region, ensuring stable and reproducible placement during surgery. The semicircular holes indicated by the black arrows on the guide plate represent the fiducial registration points for navigation. These fiducial markers are evenly distributed across the corners of the guide plate to ensure spatial stability and accurate alignment.

For the guide plate group, the virtual plan was exported to Geomagic Studio for the design of individualized surgical guides. The plates were anatomically contoured to fit the zygomatic arch and temporal bone, integrating two osteotomy slots precisely aligned with the surgical plan and three hemispherical fiducial markers (1 mm diameter) designed for optimal visibility and probe access during navigation. The guide plates were fabricated using high-precision stereolithographic 3D printing with biocompatible resin. Each plate was validated on a patient-specific 3D-printed skull model preoperatively to ensure anatomical conformity and registration accuracy. Positional tolerances were adjusted within 0.5 mm to ensure a snap-fit design (Figure 2).

### Surgical procedure and navigation workflow

**Figure 3:**
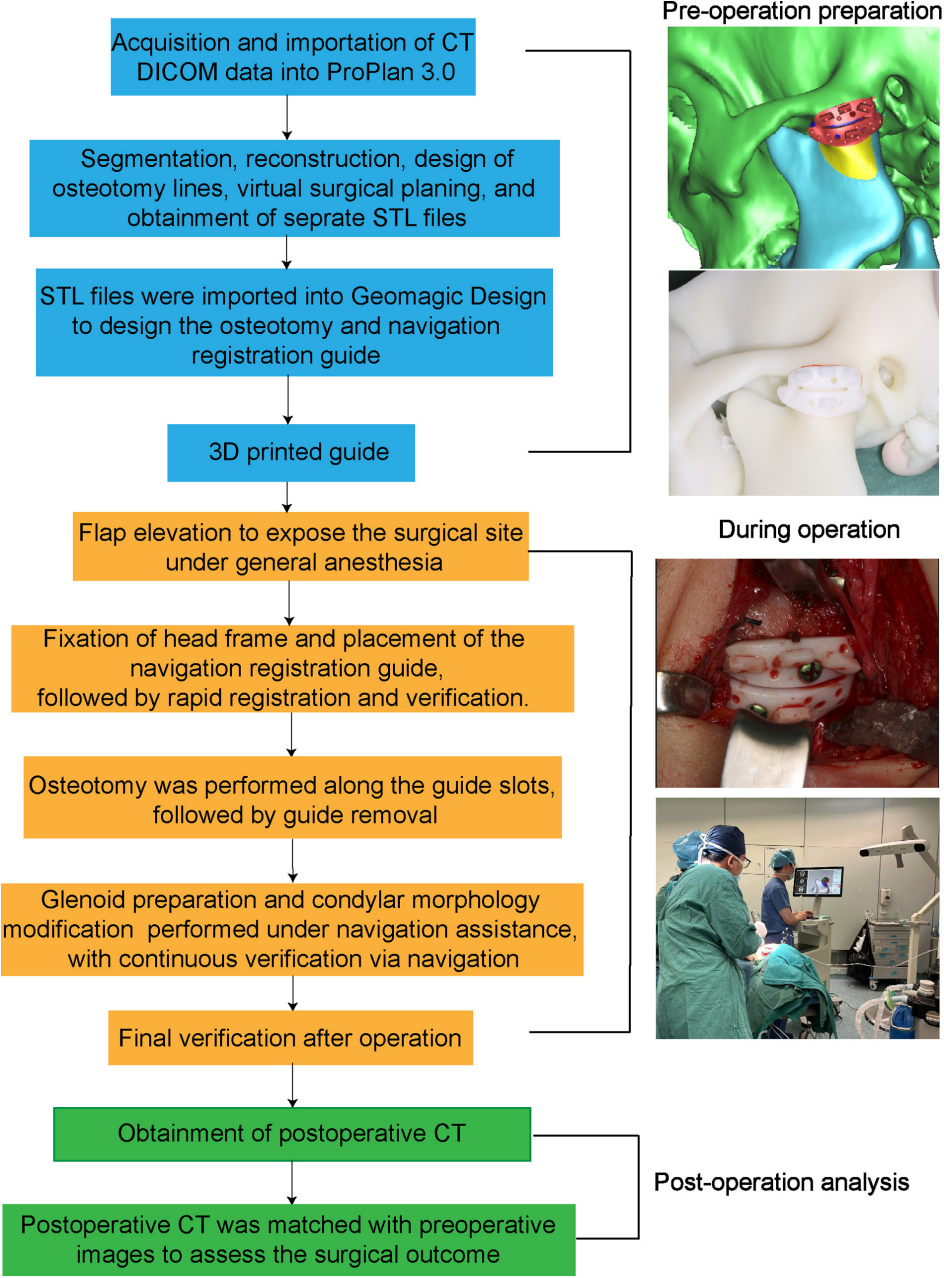
Flowchart illustrating the steps of navigation-assisted surgery using the registration guide.

All surgeries were performed under general anesthesia with nasotracheal intubation. A standard preauricular incision was made to access the TMJ region. A dynamic reference frame (DRF) was rigidly fixed to the patient's forehead using a carbon-fiber halo. The AccuNavi-A navigation system was used in both groups, and navigation accuracy was verified by calibration to pre-labeled bony landmarks. All surgical procedures were performed by the same senior attending surgeon with over 5 years of experience in TMJ surgery, to minimize inter-operator variability and ensure procedural consistency and the supporting surgical and technical team remained consistent throughout the study period (Figure 3).

In the guide plate group, the guide plate was placed and fixed with titanium mini-screws. Registration was accomplished by touching the navigation probe to the embedded fiducial spheres, following a predetermined sequence to establish spatial correlation between the patient's anatomy and the preoperative CT model. The registration process required approximately one minute per patient, and successful registration was confirmed through visual and numeric system feedback. In the dental registration group, intraoperative surface scanning of the maxillary and mandibular dentition was performed using a stereo-triangulation probe. The scanned surface was aligned to the CT-derived 3D dental model using best-fit surface matching. This process took 12–16 min per patient and was more vulnerable to deviations due to probe angle, continuous determination of cusp positions and dental feature locations, and miscommunication between the technician and the operating surgeon during the registration.

**Figure 4:**
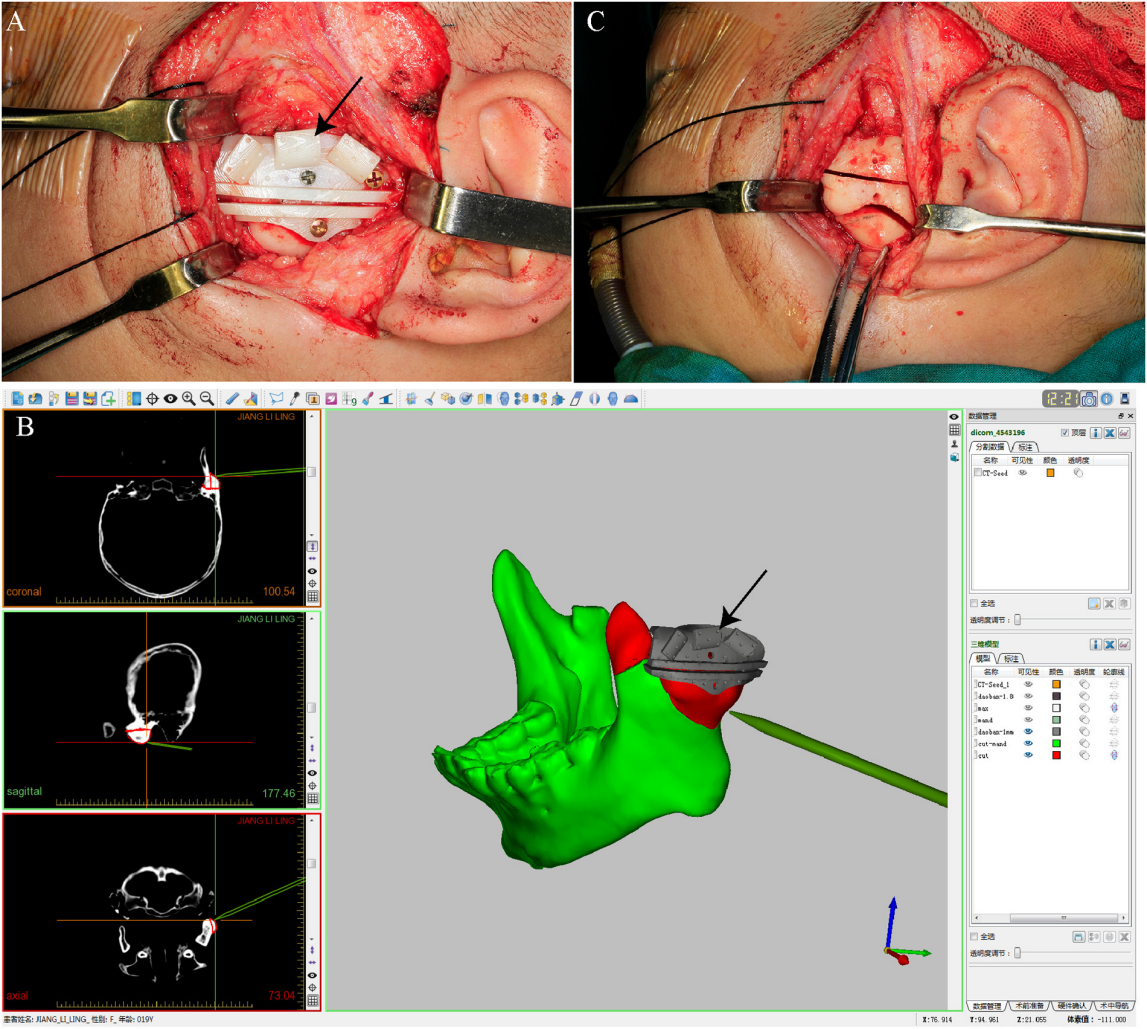
Registration and osteotomy execution during gap arthroplasty using a navigation-assisted guide system. **(A)** Fixation of the patient-specific guide plate onto the lateral mandibular ramus for stable intraoperative positioning; **(B)** Intraoperative registration aligning anatomical landmarks with the virtual plan via fiducials on the guide plate; **(C)** Osteotomy performed along the predesigned groove, enabling bone resection according to the virtual plan. The black arrows indicate the intraoperative photograph of the printed guide plate shown in Figure 2.

Osteotomy of the ankylosed condylar segment was then performed. In the guide registration group, the surgical drill was guided through the plate-defined slots for precise execution, with continuous navigation tracking verifying alignment with the preoperative plan. In both groups, the glenoid fossa was reshaped, and an interpositional barrier (either preserved disc or temporalis myofascial flap) was inserted. Hemostasis was achieved with bipolar coagulation and gelfoam placement. The wound was closed in layers with resorbable sutures. coronoidectomy on the contralateral side is carried out when indicated, in order to mitigate the risk of postoperative trismus or limited mandibular mobility. Postoperative dressing and drain placement were standardized across all cases (Figure 4).

### Outcome measurements

The primary endpoints were intraoperative registration time (in seconds) and target registration error (TRE, in mm), defined as the Euclidean distance between the actual location of a tracked probe and its virtual counterpart. TRE was calculated and documented in the software of AccuNavi-A. Secondary outcomes included total surgical duration (in minutes), mouth opening at three months (interincisal distance in mm), and incidence of intraoperative or postoperative complications (e.g., hemorrhage, nerve injury, infection). Reproducibility in this study primarily reflects intraoperative registration consistency and variance in operative time, rather than inter-operator variation, as all surgeries were performed by a single operator. All measurements were performed by independent assessors blinded to group allocation to minimize observation bias (15).

### Statistical analysis

Statistical analysis was performed using SPSS version 22.0 (IBM Corp., Armonk, NY, USA). Continuous variables were reported as mean ± standard deviation and compared using independent samples t-tests. A *P*-value < 0.05 was considered statistically significant. Data integrity was independently validated by two senior surgeons not involved in operative procedures. Outliers were assessed and removed only after dual confirmation.

This structured and multi-step workflow: spanning virtual design, surgical execution, and *post hoc* analysis, allowed for rigorous and reproducible assessment of the accuracy, safety, and workflow benefits conferred by guide plate-assisted navigation in TMJ ankylosis surgery.

## Results

All 30 patients successfully underwent TMJ gap arthroplasty without experiencing any major intraoperative or postoperative complications, underscoring the procedural safety and robustness of both surgical approaches. The absence of adverse events, such as hemorrhage, dural injury, or facial nerve trauma, demonstrates that both systems are clinically safe for use in complex temporomandibular surgeries.

At the three-month postoperative follow-up, the mean maximal interincisal mouth opening was 31.5 ± 4.23 mm in the guide plate group and 31.33 ± 3.34 mm in the dental registration group, with no statistically significant difference (P  =  0.905) (mean difference: 0.17 mm, 95% CI: −2.47 to 2.81, *P* = 0.905). This comparable functional outcome suggests that both methods effectively restore mandibular mobility and joint space.

However, a comparative analysis of intraoperative metrics reveals that the guide plate group significantly outperformed the dental registration group in multiple key domains. The mean registration time was drastically reduced in the guide plate group (56.23 ± 11.33 s) compared to the dental group (935.03 ± 85.40 s; *P* < 0.001) (mean difference: −878.8 s, 95% CI: −921.1 to −836.5, *P* < 0.001), reflecting a 16-fold improvement in registration efficiency (Table 1). This considerable time savings reduces the duration of general anesthesia, enhances intraoperative workflow, and minimizes patient exposure to potential procedural stress.

**Table 1:** Distribution of baseline characteristics and compares key surgical and postoperative variables between the guide registration group and the dental registration group.

Characteristics	Guide Registration (*n* = 12)	Dental Registration (*n* = 18)	*P*
Age (years)	33.91 ± 6.60	33.06 ± 8.82	0.779
Sex			
Male	5 (41.67%)	14 (77.78%)	0.063
Female	7 (58.33%)	4 (22.22%)	
Ankylosis Side			
Left	7 (58.33%)	9 (50%)	0.654
Right	5 (41.67%)	9 (50%)	
Registration Time (s)	56.23 ± 11.33	935.03 ± 85.40	<0.001
TRE (mm)	0.69 ± 0.10	2.82 ± 0.45	<0.001
Mouth Opening (mm) after 3months	31.50 ± 4.23	31.33 ± 3.34	0.905
Surgery Time (min)	124.63 ± 5.39	134.31 ± 12.76	0.009

Moreover, target registration error (TRE), a critical measure of spatial alignment accuracy, was significantly lower in the guide plate group (0.69 ± 0.10 mm) vs. the dental group (2.82 ± 0.45 mm; *P* < 0.001). The clinical implications of this result are substantial: achieving sub-millimeter precision reduces the likelihood of surgical deviation, particularly in anatomically dense and vulnerable regions surrounding the TMJ. Lower TRE translates directly into reduced operative risk, better protection of vital structures, and more predictable outcomes.

In terms of operative duration, the guide plate group had a shorter average surgery time (124.63 ± 5.39 min) than the dental registration group (134.31 ± 12.76 min; *P* = 0.009) (mean difference: −9.68 min, 95% CI: −17.06 to −2.30, *P* = 0.009). This time reduction is clinically significant in the context of operating room resource management and may contribute to increased surgical throughput in high-volume institutions. Notably, the variance in operative time was markedly lower in the guide plate group, indicating higher procedural reproducibility and less intraoperative variability (Table 1).

An analysis of the ankylosis side distribution revealed a near-even laterality: 58.3% of cases in the guide plate group and 50% in the dental group involved the left TMJ (*P* =  0.654). This balance eliminates lateralization as a confounding factor in comparing outcomes between groups. Additionally, baseline demographics, including age and sex distribution, were comparable, further validating the internal consistency of the study design.

**Figure 5:**
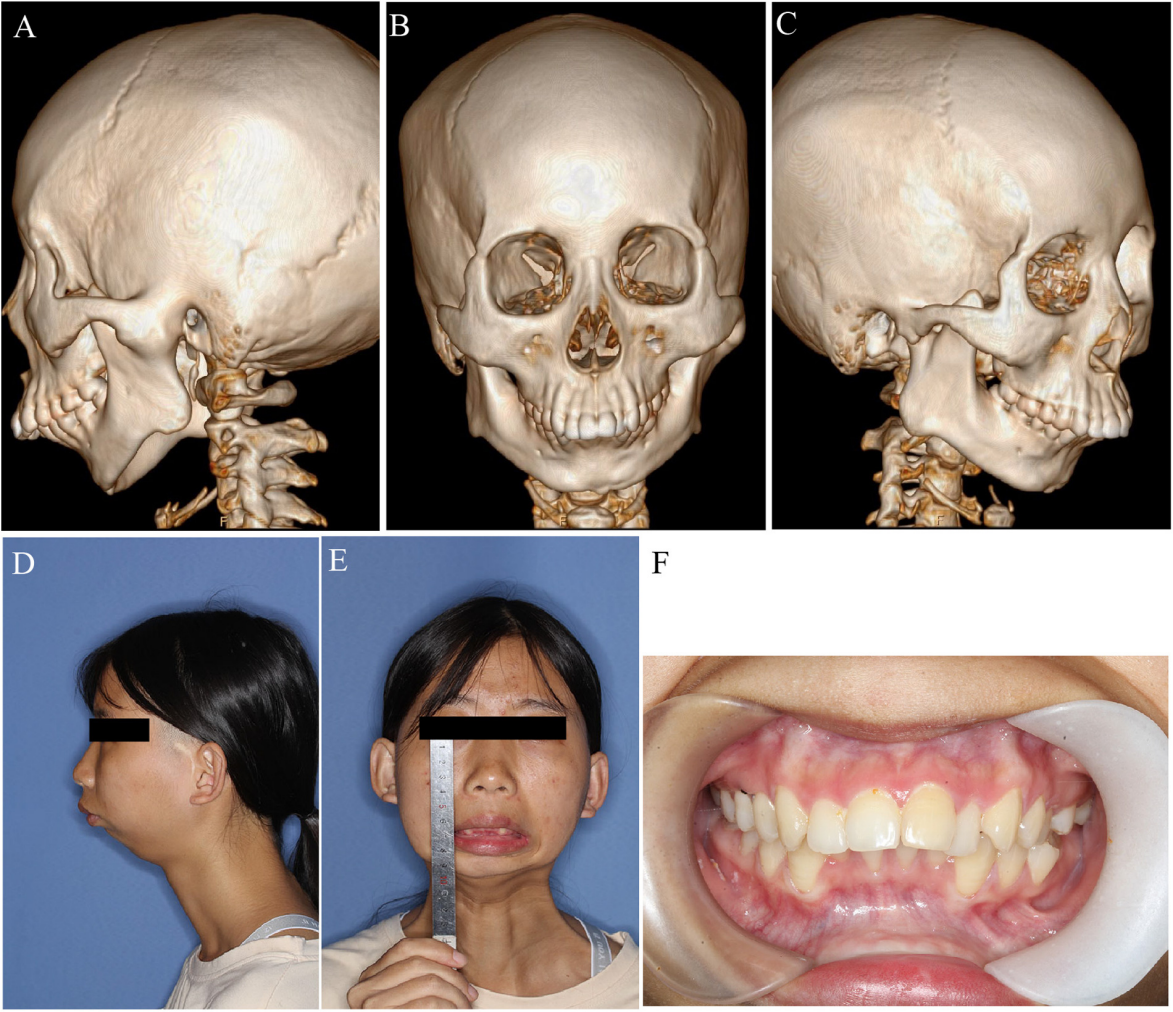
Preoperative profile of a 21 years old female patient with L TMJ ankylosis. **(A)** Left lateral CT view showing bony ankylosis; **(B)** Frontal CT view; **(C)** Right lateral CT view; **(D)** Left lateral view of facial profile; **(E)** Frontal view showing complete absence of mouth opening; **(F)** Intraoral image demonstrating complete trismus with no interincisal opening.

**Figure 6:**
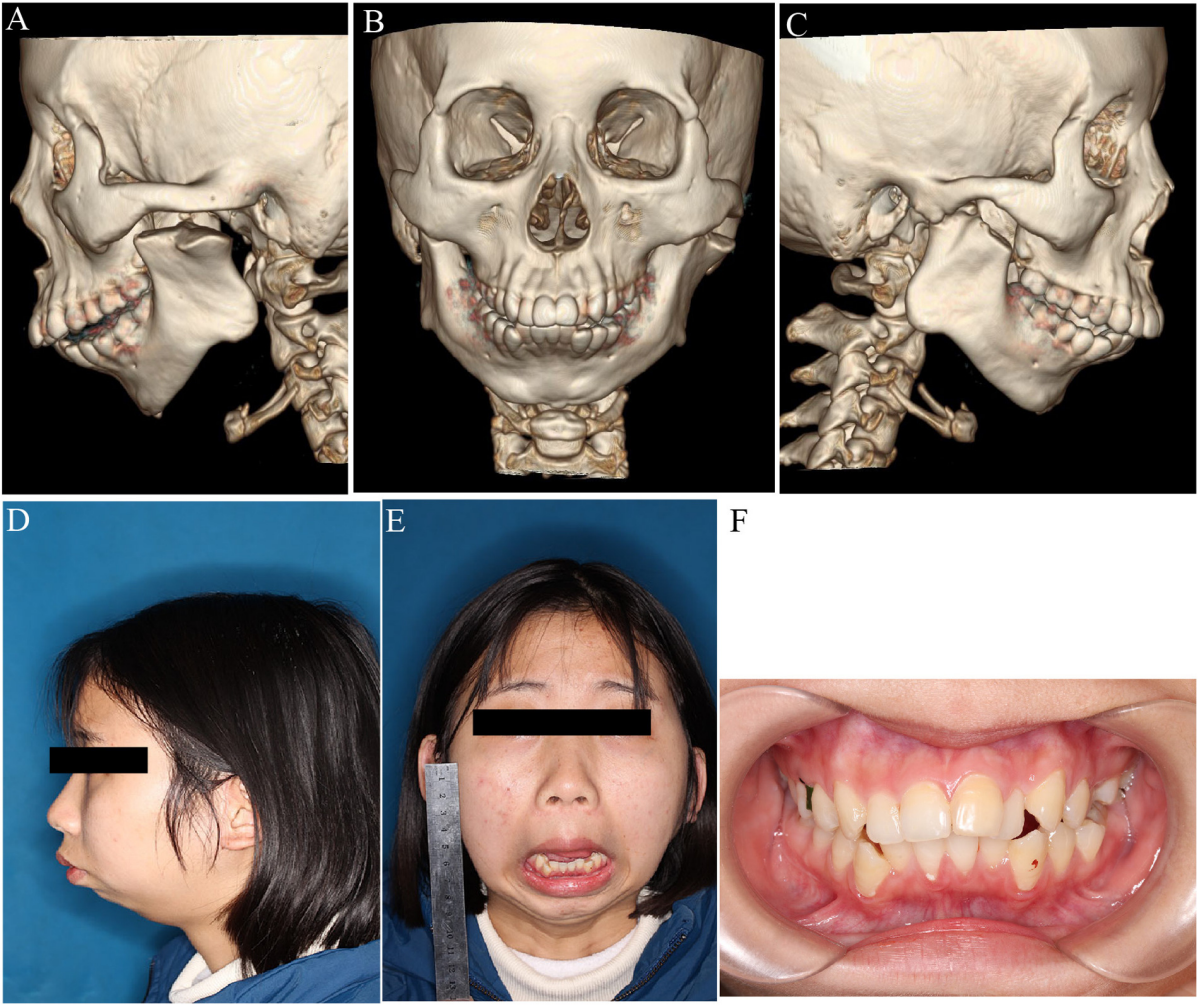
Postoperative evaluation of mandibular morphology and function. **(A–C)** 3D CT reconstructions after arthroplasty showing structural modifications; **(D,E)** Preoperative lateral and frontal facial views; postoperative maximum interincisal opening (MIO) measured at 3.5 mm; **(F)** Intraoral image showing occlusal relationship postoperatively.

Collectively, these findings provide strong quantitative evidence that the individualized guide plate system significantly enhances intraoperative efficiency and accuracy while maintaining clinical safety and functional outcomes. The technology is associated with reduced surgical duration, improved navigation fidelity, and lower risk of inadvertent injury, all of which support its adoption in routine TMJ arthroplasty practice (Figures 4–6).

## Discussion

This study provides compelling evidence for the integration of individualized osteotomy and registration guide plates into TMJ gap arthroplasty. The results demonstrate a significant enhancement in both surgical precision and efficiency, driven by several key features of the guide plate system: proximity of fiducial markers to the surgical site, anatomically conforming osteotomy pathways, and the dual functionality of physical guidance and digital registration.

The guide plate group demonstrated a notable reduction in target registration error compared with conventional dental-based registration. Achieving a mean TRE of 0.69 mm suggests improved spatial fidelity during navigation, which is particularly important in TMJ surgery, where the surgical field is adjacent to critical structures such as the dura mater and internal maxillary artery, which is critical in TMJ surgery due to the close proximity of vital structures including the dura mater, middle cranial fossa, and internal maxillary artery. In contrast, conventional dental based registration, although noninvasive, remains vulnerable to geometric error amplification due to the increased fiducial-target distance (16, 17). This observation aligns with previously reported geometric error propagation in image guided neurosurgical and craniofacial procedures, where small positional deviations may influence surgical accuracy (6, 11, 18).

In addition to enhanced accuracy, the guide plate system reduced registration time and overall operative duration. These intraoperative efficiencies may help simplify surgical workflow and reduce patient time under general anesthesia. Although modest in absolute terms, such time savings could become meaningful in high-volume clinical settings. The reduction in variance of operative time also suggests greater consistency in execution across cases. The guide plate system also demonstrated improved consistency in operative duration, reflecting greater procedural reproducibility under standardized conditions. In this study, all surgeries were performed by a single senior surgeon, and the reduced variability may reflect not only the device's guidance function but also its ability to reduce intraoperative decision-making steps. Although these results appear encouraging, further investigation is needed to determine whether similar outcomes can be achieved across different surgeons. Additionally, the guide plate eliminates the need for separate registration equipment or repeated probe calibration, thereby simplifying the surgical workflow.

The integration of preoperative planning with mechanical registration through a single device helped streamline the intraoperative workflow. This design reduces the need for repeated calibration or complex surface scanning, which can introduce time delays or inconsistencies during surgery. Simplifying these steps may be particularly valuable in resource limited environments or in surgeries requiring high precision.

From a broader perspective, this technology aligns with emerging trends in precision medicine and personalized surgery (19). The use of patient-specific anatomical data to fabricate individualized guide plates ensures optimal anatomical conformity, enhances surgical ergonomics, and minimizes the learning curve (20). More importantly, it supports the movement toward patient-tailored surgical solutions that not only improve accuracy but also adapt to unique anatomical variations. Future expansions may involve adapting this platform for use in bilateral TMJ ankylosis, cranial base reconstruction, or pediatric deformities, where small surgical corridors demand extreme accuracy. Integration with advanced imaging modalities, such as intraoperative cone-beam CT or augmented reality overlays, may further enhance spatial awareness and decision-making during critical surgical phases.

Comparatively, guide plate-assisted navigation may offer advantages over fully optical marker-based systems, particularly in dynamic surgical environments. Unlike external marker arrays, which may shift during retraction or repositioning, bone-anchored plates provide a stable registration reference throughout the procedure. This stability is especially pertinent in TMJ surgery, where anatomical access is constrained and intraoperative manipulation is frequent. The final point to emphasize is that the cost associated with guide plate fabrication is negligible relative to the substantial intraoperative benefits it confers in terms of surgical precision and efficiency. Notably, all procedures in this study were conducted without additional financial burden to patients, as they were fully supported by institutional research funding. The average turnaround time from image acquisition to finalized guide fabrication was approximately 48–72 h, and the funded cost of fabrication using in-house 3D printing facilities ranged from 300–500 RMB (∼45–70 USD) for each patient.

Limitations of the study include the single-center design and relatively small sample size, which, while sufficient for pilot analysis, call for validation in multicenter trials (21). Furthermore, a cost-benefit analysis comparing guide plate fabrication to long-term surgical efficiency gains would further substantiate the clinical value of this approach. Consideration must also be given to the preoperative design and manufacturing time associated with the guide plates, although rapid prototyping technologies are increasingly reducing this barrier (22).

## Conclusions

This study highlights the potential of using patient-specific guide plates to improve the precision and efficiency of TMJ gap arthroplasty. The approach offers a practical solution to the limitations of traditional registration methods, with favorable accuracy and workflow performance in a single-center setting. Further investigation in multicenter trials and among different surgeons will be necessary to confirm its broader clinical utility.

## Data Availability

The raw data supporting the conclusions of this article will be made available by the authors, without undue reservation.
